# Associations between maternal exposure to ambient air pollution and very low birth weight: A birth cohort study in Chongqing, China

**DOI:** 10.3389/fpubh.2023.1123594

**Published:** 2023-03-07

**Authors:** Wenzheng Zhou, Xin Ming, Yunping Yang, Yaqiong Hu, Ziyi He, Hongyan Chen, Yannan Li, Jin Cheng, Xiaojun Zhou

**Affiliations:** ^1^Department of Quality Management Section, Women and Children's Hospital of Chongqing Medical University, Chongqing, China; ^2^Department of Quality Management Section, Chongqing Health Center for Women and Children, Chongqing, China; ^3^Institute of Toxicology, College of Preventive Medicine, Army Medical University (Third Military Medical University), Chongqing, China

**Keywords:** very low birth weight, air pollution, risk assessment, environmental exposure, China

## Abstract

**Introduction:**

There have been many researches done on the association between maternal exposure to ambient air pollution and adverse pregnancy outcomes, but few studies related to very low birth weight (VLBW). This study thus explores the association between maternal exposure to ambient air pollutants and the risk of VLBW, and estimates the sensitive exposure time window.

**Methods:**

A retrospective cohort study analyzed in Chongqing, China, during 2015–2020. The Generalized Additive Model were applied to estimate exposures for each participant during each trimester and the entire pregnancy period.

**Results:**

For each 10 μg/m^3^ increase in PM_2.5_ during pregnancy, the relative risk of VLBW increased on the first trimester, with RR = 1.100 (95% CI: 1.012, 1.195) in the single-pollutant model. Similarly, for each 10 μg/m^3^ increase in PM_10_, there was a 12.9% (RR = 1.129, 95% CI: 1.055, 1.209) increase for VLBW on the first trimester in the single-pollutant model, and an 11.5% (RR = 1.115, 95% CI: 1.024, 1.213) increase in the multi-pollutant model, respectively. The first and second trimester exposures of NO_2_ were found to have statistically significant RR values for VLBW. The RR values on the first trimester were 1.131 (95% CI: 1.037, 1.233) and 1.112 (95% CI: 1.015, 1.218) in the single-pollutant model and multi-pollutant model, respectively; The RR values on the second trimester were 1.129 (95% CI: 1.027, 1.241) and 1.146 (95% CI: 1.038, 1.265) in the single-pollutant model and multi-pollutant model, respectively. The RR of O3 exposure for VLBW on the entire trimester was 1.076 (95% CI: 1.010–1.146), and on the second trimester was 1.078 (95% CI: 1:016, 1.144) in the single-pollutant model.

**Conclusion:**

This study indicates that maternal exposure to high levels of PM_2.5_, PM_10_, NO_2_, and O_3_ during pregnancy may increase the risk of very low birth weight, especially for exposure on the first and second trimester. Reducing the risk of early maternal exposure to ambient air pollution is thus necessary for pregnant women.

## 1. Introduction

Nowadays, considerable literatures on epidemiology and clinical medicine has reported the association and adverse effects of ambient air pollution on adverse pregnancy outcomes (1, 2). Low birth weight (LBW), defined as weight at birth <2,500 g, is a major neonatal adverse outcome that is strongly related to infant mortality and even producing adverse effects on children's health in adulthood (3). Very low birth weight (VLBW) is defined as weight at birth <1,500 g (4). Many researchers have explored the association between maternal exposure to ambient air pollution and low birth weight or preterm birth (5–8). Among these significant results, it has been suggested ambient air pollution may increase the risk of low birth weight. However, few studies have yet specifically focused on the association between ambient air pollution and very low birth weight. Probably because of the low incidence of VLBW, these studies can be limited by sample size (9). However, further specific research is still essential to explore the ongoing risk of air pollutants on very low birth weight.

Many scholars in China have studied and published the relationship between exposure to air pollutants and birth outcomes (10–12); however, the association between exposure to ambient air pollutants and very low birth weight has been rarely reported. Chongqing is the largest municipality in China and is located along the Yangtze River. It is a huge industrial city with 40 districts and a permanent population of 31 million. From 2014 to the present, the air quality of Chongqing has greatly improved through years of efforts by local governments. These special air quality change trends in Chongqing offer a unique research environment for studying the effects of air pollution exposure on birth outcomes that is quite different from the environments found in Europe, America, Africa, and other countries and regions.

Given that few studies have explored the potential relationship between ambient air pollution and VLBW, this study sought to estimate the association between maternal exposure to ambient air pollutants (PM_2.5_, PM_10_, SO_2_, O_3_, NO_2_, and CO) and the risk of VLBW in Chongqing, China. In doing so, it focused on the sensitive exposure time window of air pollutants for VLBW with a large sample size and also *via* precise individual exposure assessment.

## 2. Materials and methods

### 2.1. The study population

Research data for this study was gathered from a large retrospective cohort of live births from 2015 to 2020, in Chongqing, China, which was consistent with our previously published paper (13), all birth data were extracted from the birth certificate system database for Chongqing. This database contains maternal age, maternal residence address, date of birth, birth weight, gestational age, etc. We only used part of this information for our scientific research.

To facilitate a comparison of this study to previous studies, the subset of births used for this analysis was limited to singleton live births among women with 20–42 completed gestation weeks. We used the date of birth and gestational age to establish the start and end dates of gestational exposure and estimate the exposure time during the entire pregnancy and each trimester. Trimesters were defined as the 1–13, 14–27, and 28 weeks until birth (14). Cases were excluded if they had missing data for birth outcome variables. We also excluded births for any of the following: Extremely low birth weight where the value was <500 g; and a multi-fetal gestation; the mother lived ≥10 km from the nearest monitor station; and exposure data were not available for all three trimesters.

This study was approved by the Institutional Ethical Committee Board of the Chongqing Health Center for Women and Children.

### 2.2. Assessment of air pollution exposure

All ambient air pollutant concentrations, including PM_2.5_, PM_10_, SO_2_, CO, NO_2_, and O_3_, were obtained from the Chinese National Urban Air Quality Monitoring Platform (https://air.cnemc.cn:18007/) for 17 ground-based monitoring stations in nine main districts of Chongqing, China, from January 1, 2015, to December 31, 2020.

Air pollution exposure assessment was carried out using the same method as our published paper (13) mentioned above. Based on the detailed residence address of every researched pregnant woman and the location of air monitoring stations, we calculated the distance between each maternal residence and the monitoring sites using ArcGIS (version 10.2). The benefit from this process is that we were able to assign exposure values at an individual level, rather than compiling only our distinct-level measurements from the raw data (11). The proximity principle from the nearest air quality monitoring stations was applied with a cut-off distance of 10 km, which is consistent with the related research literatures (15, 16). The pregnancy exposure time started with the date of conception, according to the date of the gestational week and the last menstruation of the individual woman (17).

Daily average relative humidity and temperature were available from the China Greenhouse Data Sharing Platform (http://data.sheshiyuanyi.com). Input of any missing data was done using multiple linear interpolation based on other monitoring values.

### 2.3. Statistical analysis

To evaluate the association between ambient air pollutant exposure and the risk of VLBW in each exposure period, we performed a Generalized Additive Model (GAM), also consistent with our previously published paper (13). The effects were examined for both single-pollutant and multiple-pollutant models. The single-pollutant model was adjusted for mean temperature and humidity, the age of the mother and father, week of gestational age; further, the multi-pollutant model was adjusted for covariates that included mean temperature and humidity, age of the mother and father, week of gestational age, and additionally adjusted for other air pollutant exposure. The basic model can be described as follows:


$$\begin{array}{l} Log\left[E\left(Y_{t}\right)\right]=\alpha +\beta Z_{t}+S\left(time,df\right)+S\left(temperature,df\right)\\ \;+S\left(relativehumidity,df\right)+as.factor(Dow) \end{array}$$

where *Log*[] is a link function; t is the observation day; α is the model intercept; β is the factor for each pollutant; *Y*_*t*_ is the concentration of pollutants in day t; *S*() is the natural spline function; and Dow is dummy variable for day of week; *S*(*time, df*) is the conception time.

We estimated attributable risk percent (ARP) to explore the increased risk of VLBW caused by exposure to air pollutants. ARP indicates that the air concentration in Chongqing is higher than the national standard concentration. The standard leads to an increased proportion of VLBW occurring, which is Proportion of increased risk attributed to higher concentrations of air pollutants. In order to facilitate calculation and calculation of confidence interval, Levin's formula is applied, and the formula is as follows:


$$\begin{array}{l} ARP=\frac{P_{e}\times \left(RR-1\right)}{P_{e}\times \left(RR-1\right)+1}\times 100\% \end{array}$$

In the formula, *P*_*e*_ is the incidence of very low birth weight in Chongqing at present when the air pollutant exposure is higher than the national level I standard concentration. *RR* means that the air concentration in Chongqing is higher than the national level I standard concentration due to this study.

Sensitivity analysis were undertaken by changing the degree of freedom (df) for the time (6–8 df/year) by minimizing the Akaike information criterion (18). Finally, we selected the df of time, temperature, and relative humidity in the spline function, which were 7, 3, and 3 in the model, respectively. The GAM models were employed using R software (Version 4.1.0) with the “splines” and “mgcv” packages.

## 3. Results

### 3.1. Descriptive statistics of the research objects

In this study, a total number of 572,106 mother-newborn pairs were finally analyzed. The descriptive summary of the general characteristics of live birth data is shown in Table 1. The ages of the mothers ranged from 18 to 37 years, with an average age of 28.84 ± 4.95. The mean gestational age was 38.73 ± 1.49 weeks. Among them, 24,497 (4.28%) were LBW and 1,725 (0.3%) were VLBW.

**Table 1:** Descriptive summary of the general characteristics of live birth data.

**Variables**	**LBW**	**VLBW**	**Non-VLBW**	**Total**	***p-*value**
	**(*n* = 24,497, 4.28%)**	**(*n* = 1,725, 0.3%)**	**(*n* = 570,381, 99.7%)**	**(*n* = 572,106)**	
**Gestational age**	35.15 ± 2.61	30.02 ± 2.55	38.75 ± 1.41	38.73 ± 1.49	<0.001
**Preterm birth**					
Yes	16,421 (67.01%)	1,706 (98.90%)	31,961 (5.60%)	33,667 (5.88%)	<0.001
No	8,086 (32.99%)	19 (1.10%)	538,420 (94.40%)	538,439 (94.12%)	
**Maternal age**	29.58 ± 5.15	30.18 ± 5.16	28.84 ± 4.95	28.84 ± 4.95	<0.001
<20 years	416 (1.7%)	22 (1.28%)	8,184 (1.43%)	8,206 (1.42%)	<0.001
20–24 years	3,589 (14.65%)	203 (11.77%)	97,075 (17.02%)	97,278 (17.11%)	
25–29 years	8,536 (34.85%)	578 (33.51%)	229,341 (40.21%)	229,919 (40.43%)	
30–34 years	7,837 (31.99%)	575 (33.33%)	163,020 (28.58%)	163,595 (28.44%)	
≥35 years	4,092 (16.7%)	346 (20.06%)	72,265 (12.67%)	72,611 (12.51%)	
Missing	27(0.11%)	1 (0.05%)	496 (0.08%)	497 (0.08%)	
**Father age**	31.90 ± 5.96	32.76 ± 5.98	31.13 ± 5.62	31.14 ± 5.62	<0.001
<20 years	76 (0.31%)	4 (0.23%)	1,736 (0.30%)	1,740 (0.30%)	<0.001
20–24 years	1,842 (7.52%)	77 (4.46%)	48,907 (8.57%)	48,984 (8.56%)	
25–29 years	7,021 (28.66%)	423 (24.52%)	191,604 (33.59%)	192,027 (33.57%)	
30–34 years	8,101 (33.07%)	563 (32.64%)	183,848 (32.23%)	184,411 (32.23%)	
≥35 years	6,702 (27.36%)	525 (30.43%)	132,639 (23.25%)	133,164 (23.28%)	
Missing	755 (3.08%)	133 (7.71%)	11,647 (2.04%)	11,780 (2.05%)	
**Conception season**					
Spring	5,850 (23.88%)	416 (24.12%)	134,931 (23.66%)	135,347 (23.66%)	<0.001
Summer	6,062 (24.75%)	411 (23.83%)	135,916 (23.83%)	136,327 (23.83%)	
Autumn	6,324 (25.82%)	448 (25.97%)	154,892 (27.16%)	155,340 (27.15%)	
Winter	6,261 (25.56%)	450 (26.09%)	144,642 (25.36%)	145,092 (25.36%)	

The *p*-value stands for the comparison of VLBW and non-VLBW.

### 3.2. Air pollutants descriptive statistics

The characteristics of air pollution and their meteorological factors are summarized in Table 2. The mean concentration of PM_2.5_ during a whole pregnancy was 41.62 μg/m^3^, and the mean concentration of PM_10_ at the same time was 66.39 μg/m^3^. The mean concentrations were 38.95 μg/m^3^ for NO_2_, 1.02 mg/m^3^ for CO, 9.73 μg/m^3^ for SO_2_, 38.45 μg/m^3^ for O_3_, 20.25°C for the apparent mean temperature, and 75.25% for relative humidity during the entire study period.

**Table 2:** Descriptive summary of air pollutants and meteorological factors in the study area.

**Pollutants (μg/m^3^)**	**Mean**	**SD**	**Min**	**Max**	**Percentiles**		
					**25th**	**50th**	**75th**
PM_2.5_	41.62	10.01	17.82	83.65	34.39	42.69	47.50
PM_10_	66.39	12.61	28.79	121.46	59.12	67.30	73.66
NO_2_	38.95	6.46	10.78	68.19	35.50	38.35	41.15
CO (mg/m^3^)	1.02	0.18	0.54	1.52	0.89	1.01	1.14
SO_2_	9.73	3.14	3.21	22.11	7.32	9.12	11.27
O_3_	38.45	13.34	8.27	105.65	30.24	39.07	47.38
Temperature (°C)	20.25	2.73	13.13	29.09	18.43	20.42	21.86
Relative humidity (%)	75.25	2.17	66.89	80.37	73.85	75.21	76.81

Period for January 1, 2015, to December 31, 2020.

**Figure 1:**
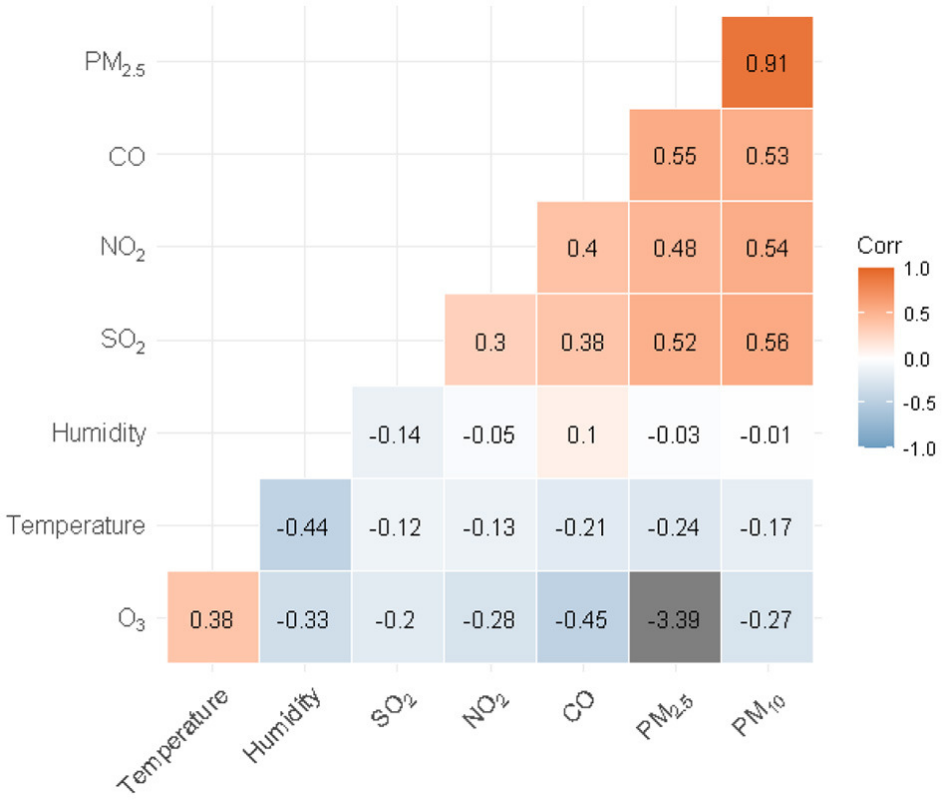
Correlation analysis results for air pollutants and meteorological factors.

The correlation between most pollutant correlations were positive except O_3_. In addition, except for O3 and temperature, CO and humidity, the correlation between other air pollutants and meteorological factors was mostly negative. A positive correlation between PM_2.5_ and PM_10_ (*r* = 0.910), and a negative correlation between PM_2.5_ and average daily temperature (*r* =-0.244) were observed. The correlation analysis results between air pollutants and meteorological factors are shown in Figure 1.

### 3.3. Associations between air pollutants and VLBW

The associations found between Air pollutants and VLBW were calculated using the GAM models. The adjusted relative risks (RRs) and corresponding 95% confidence intervals (CIs) for VLBW to maternal exposure to air pollutants by pregnancy trimester are given in Table 3.

**Table 3:** Adjusted relative risks (RRs) and corresponding 95% confidence intervals (CIs) from GAM models for VLBW maternal exposure to air pollutants by trimester of pregnancy.

**Pollutant**	**Model**	**Trimester 1**		**Trimester 2**		**Trimester 3**		**Entire pregnancy**	
		***RR***	***95% CI***	***RR***	***95% CI***	***RR***	***95% CI***	***RR***	***95% CI***
PM_2.5_	Model 1	**1.100**	**(1.012, 1.195)**	0.968	(0.883, 1.062)	1.017	(0.965, 1.072)	1.018	(0.908, 1.141)
	Model 2	1.070	(0.972, 1.176)	0.934	(0.836, 1.045)	1.041	(0.978, 1.108)	1.034	(0.891, 1.200)
PM_10_	Model 1	**1.129**	**(1.055, 1.209)**	1.048	(0.978, 1.124)	1.007	(0.970, 1.045)	1.050	(0.962, 1.145)
	Model 2	**1.115**	**(1.024, 1.213)**	1.041	(0.955, 1.135)	1.028	(0.980, 1.078)	1.100	(0.975, 1.242)
SO_2_	Model 1	1.198	(0.921, 1.562)	1.234	(0.946, 1.610)	0.961	(0.760, 1.216)	1.199	(0.878, 1.641)
	Model 2	1.062	(0.785, 1.438)	1.257	(0.928, 1.701)	0.945	(0.731, 1.222)	1.080	(0.739, 1.577)
NO_2_	Model 1	**1.131**	**(1.037, 1.233)**	**1.129**	**(1.027, 1.241)**	0.962	(0.897, 1.032)	1.069	(0.967, 1.182)
	Model 2	**1.112**	**(1.015, 1.218)**	**1.146**	**(1.038, 1.265)**	0.944	(0.872, 1.022)	1.071	(0.964, 1.190)
O_3_	Model 1	1.013	(0.952, 1.077)	**1.078**	**(1.016, 1.144)**	0.995	(0.952, 1.037)	**1.076**	**(1.010, 1.146)**
	Model 2	1.036	(0.967, 1.110)	1.069	(0.996, 1.146)	0.996	(0.952, 1.041)	1.083	(0.998, 1.175)
CO	Model 1	0.993	(0.958, 1.030)	0.974	(0.939, 1.010)	0.999	(0.971, 1.028)	0.960	(0.923, 0.998)
	Model 2	1.002	(0.962, 1.044)	0.999	(0.960, 1.041)	0.998	(0.967, 1.029)	0.987	(0.940, 1.036)

The bold face indicates statistical significance established at *p* < 0.05 in the above three models. Model 1: single-pollutant model, adjusted for covariates including mean temperature and humidity, age of mother and father, and age of gestation; Model 2: multi-pollutant model, adjusted for covariates including mean temperature and humidity, age of mother and father, weight of birth, and additionally adjusted for other air pollutants.

We observed that for each 10 μg/m^3^ increase in PM_2.5_ during pregnancy, the relative risk of VLBW increased on the first trimester, with RR=1.100 (95% CI: 1.012, 1.195) in the single-pollutant model. Similarly, for each 10 μg/m^3^ increase in PM_10_, there was a 12.9% (RR = 1.129, 95% CI: 1.055, 1.209) increased risk for VLBW on the first trimester in the single-pollutant model, and an 11.5% (RR = 1.115, 95% CI: 1.024, 1.213) increase in the multi-pollutant model, respectively.

The first and second trimester exposures of NO_2_ were found to have statistically significant RR values for VLBW. The RR values on the first trimester were 1.131 (95% CI: 1.037, 1.233) and 1.112 (95% CI: 1.015, 1.218) in the single-pollutant model and the multi-pollutant model, respectively; The RR values on the second trimester were 1.129 (95% CI: 1.027, 1.241) and 1.146 (95% CI: 1.038, 1.265) in the single-pollutant model and the multi-pollutant model, respectively.

The RR of O_3_ exposure for VLBW on the entire trimester was 1.076 (95% CI: 1.010, 1.146), and on the second trimester was 1.078 (95% CI: 1:016, 1.144) in the single-pollutant model. As shown in Table 3, No statistically significant RR was found for SO_2_ and CO in each trimester of pregnancy.

**Figure 2:**
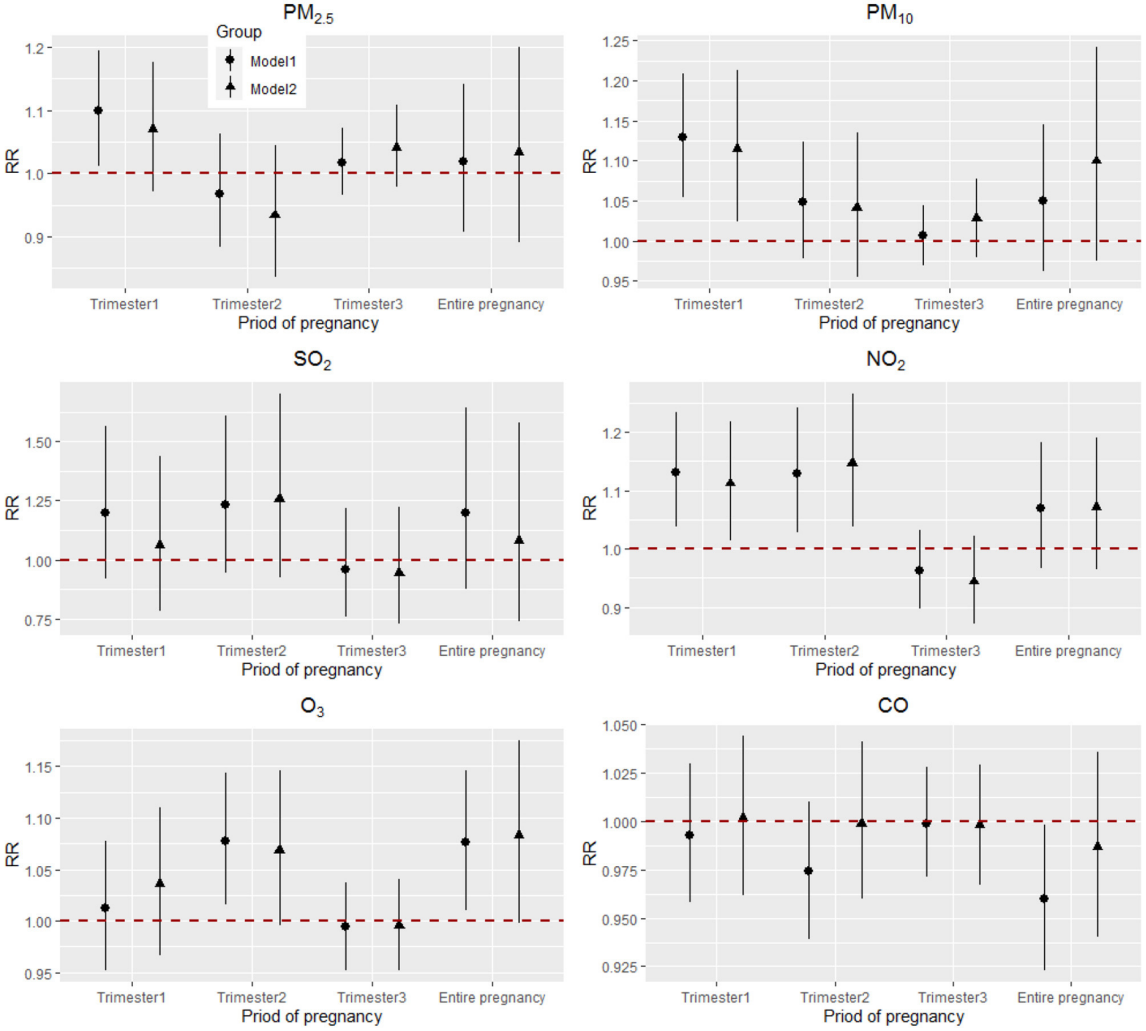
Adjusted RRs (95% CIs) for VLBW associated with air pollutants during the different stages of pregnancy in Model 1 and Model 2. Model 1: single-pollutant model, adjusted for covariates including mean temperature and humidity, age of mother and father, and age of gestation, as represented by a circle; Model 2: multi-pollutant model, adjusted for covariates including mean temperature and humidity, age of mother and father, weight of birth, and additionally adjusted for other air pollutants, as represented by a triangle.

Overall, the association with statistical significance between maternal exposure to air pollutants and VLBW was concentrated in PM2.5, PM10, and NO_2_. Exposure at different stages of pregnancy had different results, particularly the risk of early pregnancy exposure was relatively higher. Forest plots of the RR values and 95% CIs for VLBW associated with maternal exposure to six pollutants during the different stages of pregnancy are shown in Figure 2.

### 3.4. Attribution analysis of maternal exposure to air pollutants and VLBW

In this study, we calculated the attributable risk percentage (ARP) of PM_2.5_ for VLBW throughout the entire pregnancy according to the Chinese Class I Standard of PM_2.5_ < 35 μg/m^3^. We adjusted for the covariates including mean temperature and mean humidity, parental age, and gestational age. We estimated the ARP of PM_2.5_ for VLBW that was attributable to PM_2.5_ exposure concentration to be higher than the Chinese Class I Standard after adjusting for covariates. The ARP was 17.89% (95% CI: 10.5%, 24.26%). Similarly, the attributable risk percentage (ARP) of PM_10_ for VLBW was calculated using the Chinese Class I Standard of PM_10_ < 50 μg/m^3^. Lastly, the ARP of PM_10_ for VLBW was 36.81% (95% CI: 25.69%, 46.01%).

## 4. Discussion

In this study, we used a generalized additive model (GAM) to analyze the exposure-response association of air pollutants on the risk of very low birth weight. It revealed that maternal exposure to PM_2.5_ and PM_10_ in the first trimester of pregnancy was associated with increased risk of VLBW. In addition, a positive association with VLBW was linked to NO_2_ exposure during the first and second trimesters of pregnancy. These results are a valuable supplement to the few previous association researches for maternal exposure to ambient air pollution and the risk of very low birth weight (19). Especially in China, such similarly related research is quite rare.

There are a lot of studies that have demonstrated that maternal exposure to fine ambient air pollution increases the risk of preterm birth and low birth weight (2, 20–22). Ghosh et al. (23) conducted a meta-regression and analysis related association of PM_2.5_ pollution and adverse perinatal outcomes for 204 countries and territories. Its pooled estimates indicated 22 grams (95% UI: 12, 32) lower birth weight, and 11% greater risk of LBW (1.11, 95% UI: 1.07, 1.16) per 10 μg/m^3^ increment in ambient PM_2.5_. Globally, an estimated 15.6% (95% UI: 15.6, 15.7) of all LBW infants were attributable to total PM_2.5_ in 2019. A meta-analysis by Bekkar et al. (2) reported that positive associations between exposure to air pollution and LBW were found across all US geographic regions. Exposure to PM_2.5_ or ozone was associated with an increased risk of LBW in 25 of 29 studies (86%). Niu et al. (24) carried out a cohort study in Los Angeles, California, and found early pregnancy to mid-pregnancy exposures to PM_2.5_, PM_10_, and NO_2_ were associated with lower birth weight. Their result is highly consistent with our research. Still, some studies have suggested that the sensitive exposure period for NO_2_ is throughout all of a pregnancy (12, 25). Although the stages of a sensitive window to air pollution exposure varied in the different literatures, the basic consensus is that maternal exposure to air pollution, particularly during the critical windows of pregnancy, significantly do increases the risk of LBW.

Over the past decade, numerous studies have been published on air pollutant exposure and low birth weight in China (12, 26, 27). The study by Yuan et al. (28) reported critical windows of gestational exposure to PM_2.5_ were identified as 31st−34th gestational weeks for reduced birth weight, and 38th−42nd weeks for LBW, respectively. Liang et al. (29) conducted a study on 1,455,026 singleton births during 2014–2017 in nine cities of Guangdong, China, and found PM_2.5_ was significantly associated with LBW in every trimester of pregnancy stage, with stronger effects on the first and third trimester for each 10 μg/m^3^ increase in PM_2.5_ concentrations. The results of a cohort study performed in Changsha, China, further showed term LBW was significantly associated with exposure to ambient PM during pregnancy, with OR = 1.47 (95% CI: 1.00, 2.14) for per IQR increase after adjustment for the covariates and home environmental factors (3). Specifically, the authors identified a significant association in the early phase of pregnancy including conception month and the first trimester. Zou et al. (26) performed a retrospective observational study on 2,527 preschoolers in Shanghai, China, and indicated that exposure to NO_2_ was a risk factor for LBW and T-LBW. The difference when compared to this study is that effects of exposures could be greater during early periods than during later periods of gestation.

Related studies have suggested different sensitive windows worldwide. The differences may be due to study design, air pollution level, regional disparity, components of PM, and sample size, etc. However, most of the sensitive time windows are concentrated in the first trimester. For example, in a study of seven states in the U.S. (30) for associations between maternal exposure to PM_2.5_ and the risk of LBW, showed a statistically significant correlation during the entire stage of pregnancy and all specific trimesters in New York, for the full gestation, the first and third trimesters in Minnesota, and for the entire pregnancy and first trimester in New Jersey.

The current study focused on associations between maternal exposure to ambient air pollution and VLBW, which was unique to our study. We observed that the first trimester may be the sensitive window for PM_2.5_ and PM_10_, consistent with a few previous studies on LBW (29, 31). Meanwhile, the first and second trimesters constituted the susceptible exposure window for NO_2_. On the other hand, a few studies have shown different staging methods with consequently different results. For example, several researchers divided pregnancy into months or weeks to evaluate the relationship between exposure and outcomes. Our large sample size and the exposure evaluation mode of individual assessment allowed us to reliably estimate the associations. However, the effect of exposure sensitive time does need to be further explored, and personal exposure measurements need to be more widely utilized.

Few studies have evaluated the disease burdens of PM exposure on LBW worldwide. We found 17.89% (95% CI: 10.5%, 24.26%) of VLBW was attributable to a higher PM_2.5_ exposure (PM_2.5_ ≥ 35 μg/m^3^) relative to the Chinese Class I Standard after adjusting for covariates. The ARP of PM_10_ for VLBW was 36.81% (95% CI: 25.69%, 46.01%), calculated for PM_10_ exposure concentration higher than 50 μg/m^3^. These results were statistically sufficient to demonstrate the harmful effects of high PM exposure on very low birth weight. Liu et al. (4) estimated the LBW burden caused by outdoor PM_2.5_ exposure in Shanghai, China, in 2013, according to Shanghai's Class I Standard (15 μg/m^3^). Those results showed that 23.36% (95%CI: 3.86%, 40.02%) of LBW could be attributed to PM_2.5_ exposure. Our results provided a particular estimate of attribution analysis of PM exposure on VLBW in China.

There are several possible biologic mechanisms through which ambient air pollution can cause LBW (32, 33). Yet, no studies have specifically focused on the mechanisms that cause very low birth weight. Current research reports mainly include systemic oxidative stress and inflammatory response that induces premature birth; maternal endocrine disorder; the release of inflammatory factors and entering into the placenta; direct toxicity to the placenta or fetus, etc. (7, 34, 35). Studies on the effects of different components of PM_2.5_ on birth outcomes have shown that the component elements of carbon, calcium, copper, nickel, titanium, zinc, aluminum, and antimony are associated with low birth weight (36). The toxicological effects of metal components are inferred as well, mainly by increasing oxidative stress (37). The biological mechanisms related to VLBW, however, need to be further investigated and explored in the future.

This study did have some limitations. First, due to the large sample size, it was difficult to obtain comprehensive and complete information. Some potential risk factors were not considered in this study, such as maternal nutritional status, pregnancy complications, and life behaviors, genetic information, etc. These factors may have confounded the association results. However, previous similar studies have found little change in efficacy estimates based on whether or not these factors are adjusted (33). Second, as with most related studies, exposure measurement errors were inevitable. The type of area, proximity of green/blue area and the “quality” of neighborhood can also be important in assessment of exposure level. However, due to the absence of these variables in the original data, we did not conduct further analysis about this. The proximity principle from nearby air quality monitoring stations was applied to serve as the estimates of individual air pollution exposure. Moreover, we limited the exposure concentration assessment to within 10 km of the monitoring station. We did not assess the movement of pregnant women during pregnancy. Fortunately, the large sample size used for this study balanced that situation to some extent. Third, the composition of PM is complex, and that composition was not obtained and analyzed in this study. It is possible that different pollutant components can have inconsistent effects on VLBW. The risk effects caused by specific components will be explored in subsequent relevant studies.

## 5. Conclusions

In conclusion, this study provides special evidence on the associations between air pollutant exposure during pregnancy and VLBW using a retrospective birth cohort study. We estimated that maternal exposure to high levels of PM_2.5_, PM_10_, NO_2_, and O_3_ during pregnancy may increase the risk of very low birth weight. The sensitive period for that exposure window is likely to be the first and second trimesters. Reducing the risk of early maternal exposure to ambient air pollution is thus necessary for pregnant women.

## Data availability statement

The raw data supporting the conclusions of this article will be made available by the authors, without undue reservation.

## Author contributions

WZ, XM, JC, and XZ: conceptualization. XM and JC: methodology. YY: software. YY and YH: validation. ZH: formal analysis. XM and HC: investigation. WZ: resources, project administration, and funding acquisition. HC and YL: data curation. WZ and XM: writing—original draft preparation. JC and XZ: writing—review and editing. XM and ZH: visualization. JC: supervision. All authors have read and agreed to the published version of the manuscript.
